# A comparison of methods for measuring camouflaging in autism

**DOI:** 10.1002/aur.2850

**Published:** 2022-11-24

**Authors:** Benjamin Hannon, William Mandy, Laura Hull

**Affiliations:** ^1^ Cambridgeshire & Peterborough NHS Foundation Trust, Cambridge UK; ^2^ Department of Clinical, Educational & Health Psychology University College London London UK; ^3^ Centre for Academic Mental Health, Population Health Sciences Bristol Medical School, University of Bristol Bristol UK

**Keywords:** autism, camouflaging, measurement, questionnaire, reliability and validity

## Abstract

Interest in social camouflaging has led to a multiplicity of measurement methods of uncertain validity. This two‐part investigation first used a systematic review (“Study 1”) to identify and appraise methods used to quantify camouflaging of autistic traits, using the Consensus‐based Standards for the Selection of Health Status Measurement Instruments checklist. A total of 16 distinct measurement tools were identified; all are in the preliminary phases of psychometric evaluation. The systematic review highlighted: (1) the need for parent‐report tools which specifically measure camouflaging; and (2) a lack of studies looking at associations between different methods of camouflaging, which limits understanding of their validity. “Study 2” aimed to begin to address these gaps in knowledge. We created a parent‐report version of the Camouflaging Autistic Traits Questionnaire (CAT‐Q) and evaluated its concurrent validity in autistic young people by examining associations with the self‐report CAT‐Q and a discrepancy measure. Discriminant validity was investigated by comparing all three methods of measuring camouflaging to a measure of social skills, to test whether they assess a construct distinct from social ability. The self‐ and parent‐report CAT‐Q were significantly related (*r* = 0.47, 95% CI = 0.24–0.65), and were related weakly (*r* = 0.20, 95% CI = −0.06 to 0.43) and strongly (*r* = 0.46, 95% CI = 0.23–0.64), respectively, to the discrepancy approach. No measure was associated with social skills. Improving the psychometric properties of these methods, and introducing a novel parent‐report measure, may help selection of appropriate methods in future research and integration into clinical practice.

## Lay Summary

The first study looked at published research to evaluate tools used to measure camouflaging in autism; showing that there is still much to learn about whether existing measures are accurate, and that there are no parent‐report tools designed to measure camouflaging. The second study started to fill these gaps in knowledge, by developing and testing a parent‐report camouflaging measure, and comparing it to other common ways of measuring camouflaging. This showed that, for teenagers, a new parent‐report questionnaire can be useful for measuring camouflaging.

## INTRODUCTION

Since 2017, there has been a dramatic increase in research into camouflaging in autism. Camouflaging is defined here as the masking of or compensation for autistic characteristics during social interactions (Hull et al., 2017; Lai et al., 2017; Livingston & Happé, 2017). It can include conscious efforts to appear “less autistic” (Hull et al., 2017; Livingston & Happé, 2017), as well as the subconscious suppression of autistic characteristics (Lawson, 2020), potentially as the result of internal or external stigma (Pearson & Rose, 2021; Perry et al., 2021). Researchers have recently suggested that camouflaging may reflect the impression management performed across neurotypes, but with unique mechanisms and consequences within autistic populations (Ai et al., 2022). We note here that researchers and autism community members use a variety of terms to describe this phenomenon, including “masking”, “compensation”, “passing”, and “adaptive morphing.” However, we choose to use the term “camouflaging” in the present article as this is the most commonly used term in the literature at the time of writing (e.g., Ai et al., 2022; Cook et al., 2021).

This paper describes a two‐stage research process designed to yield insights into how to measure camouflaging for research and clinical practice. First, in Study 1, we conducted a systematic review to map and evaluate current methods of measuring camouflaging. Study 2 sought to fill a gap in the literature identified in Study 1, by developing a parent‐report camouflaging measure. We compared this new method of measuring camouflaging with two already‐established methods of measurement (self‐report and discrepancy), to explore concurrent validity. “Self‐report” refers to methods where individuals identify their own levels of camouflaging; “discrepancy” refers to methods where camouflaging is quantified as a discrepancy between internal and external measures of autistic characteristics, such as theory of mind and behavioral expression of autism. Also, we examined associations with a measure of social skills to evaluate discriminant validity, on the basis that camouflaging and social skills are conceptually distinct. The two studies are presented separately, followed by a brief general discussion.

## STUDY 1: SYSTEMATIC REVIEW

Several recent reviews (Cook et al., 2021; Hull et al., 2020a; Tubio‐Fungueirino et al., 2020), opinion pieces (Ai et al., 2022), and editorials (Fombonne, 2020; Mandy, 2019) have described the burgeoning field of camouflaging research. Amongst the key criticisms of the extant camouflaging literature is that: (1) findings are largely generated from internet‐recruited adult samples, with an under‐representation of young people; and (2) there is a lack of clarity about how best to operationalize and measure camouflaging (see Williams, 2021 for a more detailed critique). Of relevance to the present study, a variety of methods to measure camouflaging, often using quite different operationalizations, have been developed. These fall in to two broad categories (Cook et al., 2021; Hull et al., 2020a). Discrepancy measures seek to quantify camouflaging by evaluating the difference between a person's internal autistic state and their observable autism‐relevant characteristics (e.g., Lai et al., 2017). In contrast, observational/reflective methods seek to quantify the extent to which an individual uses specific camouflaging strategies and behavior, via self‐reflection (e.g., Hull et al., 2019) or observation (e.g., Dean et al., 2017).

The present systematic review sought to evaluate measures within both of these categories, using standardized quality appraisal criteria for the first time. The aim was to identify strengths and limitation of current measures of camouflaging in order for future research to improve the measurement of camouflaging.

### Methods

#### Eligibility criteria

Studies were included in the review if they were peer‐reviewed, written in English and reported measurement of the camouflaging of autistic traits, regardless of participants' diagnostic status. Outcome variables were required to be either quantitative and/or categorical. The first published study to evaluate a novel measure was included where multiple studies have been published on the same measure. Additional studies that have used a previously published measure are included in Table S1, alongside their evaluated rating.

#### Information sources and search strategy

Two electronic bibliographic databases were initially searched for relevant articles: PsycINFO and Web of Science. Both databases were searched from first publication to October 2020. The search was updated using Google Scholar to search for articles published between October 2020 and March 2022. The search terms included [autis*] AND [camouflag*] OR [mask*] OR [compensat*] OR [pass*]. These terms were chosen to reflect the variety of terms used in the camouflaging literature, as previously discussed. We also completed a manual search of the reference lists from fully accessed articles. Experts researching camouflaging were also contacted, and invited to suggest any additional papers that may have been missed.

#### Selection process

**FIGURE 1:**
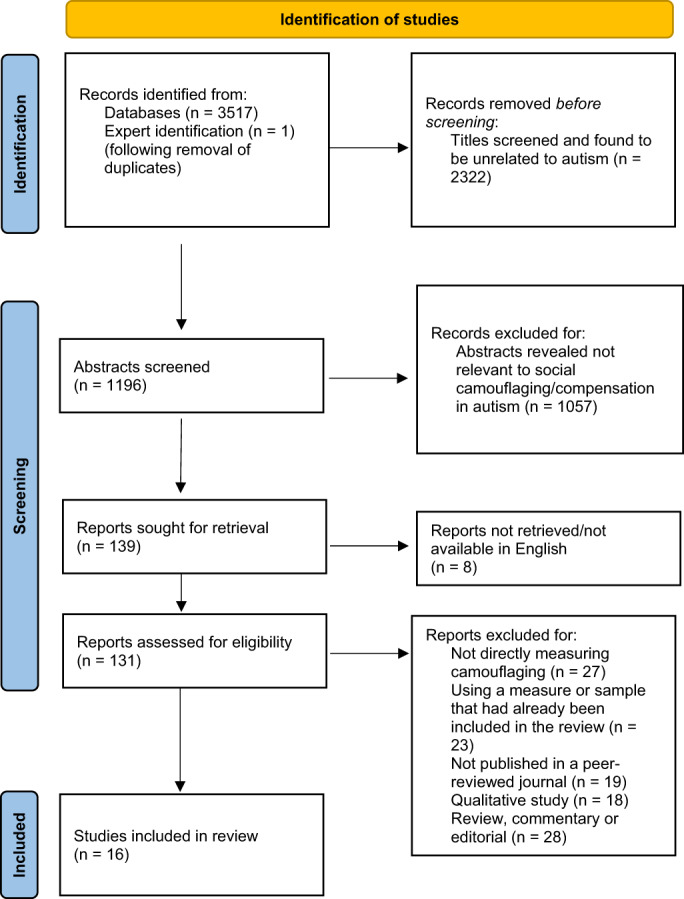
Study inclusion flow diagram highlighting three‐phase process from identification to inclusion.

To determine whether the inclusion criteria were met, all records were screened by the first author (B. H.) using a three‐phase process. The initial search returned 3517 unique articles when using the “all fields” function. As such, phase one involved searching within the title, abstract, and key words only, using the search terms previously mentioned. In phase two, the remaining titles and abstracts were examined for basic relevance to the camouflaging of autistic traits. The final phase involved accessing the remaining articles in full and including only those that met the criteria described above. Each stage is outlined in Figure 1 (below).

#### Quality appraisal and data collection process

The Consensus‐based Standard for the Selection of Health Status Measurement Instruments (COSMIN) checklist (Mokkink et al., 2010) was used to critically appraise the camouflaging measurement methods. The COSMIN checklist contains 102 total items. A total of 98 items assess the psychometric quality of the measurement instrument across nine properties (see Supporting Information S1). The checklist requires the user to only assess the properties that are reported by the authors, or those that are relevant to the measurement instrument. The current review used the updated four‐point scoring system for the COSMIN (Terwee et al., 2012). All 98 psychometric items are scored along a four‐point scale, ranging from “excellent” and “good”, through to “fair” and “poor.” The nine measurement properties subsequently receive an overall rating along the same four‐point scale, using a “worse score counts” methodology, meaning that the lowest rated item within each measurement property reflects the overall rating.

Each camouflaging tool was assessed by the first author (B. H.) by reading the associated paper twice, and subsequently rating them using the COSMIN checklist. Five papers were double coded by the first (B. H.) and senior author (L. H.) to assess inter‐rater reliability. Results indicated “substantial” agreement, *k*_w_ = 0.67. Where discrepancies arose, a consensus meeting was used to reach agreement and coding notes were updated accordingly.

Due to the fledgling nature of camouflaging research, content validity was not possible to evaluate in its entirety as there are no universally agreed criteria for all aspects of camouflaging. As such, item four from the construct validity property was omitted.

### Results

Figure 1 documents the study selection process from first identification, through the previously mentioned three‐phase screening process. After screening the abstracts, 139 studies were deemed to have basic relevance to camouflaging. A total of 16 papers describing unique camouflaging measurement tools met the inclusion criteria.

#### Study characteristics

Table 1 lists the studies included in the current review. Measurement tools that have not been formally named will be referred to by the lead author's name from the original publication. From the 16 included studies, three of the identified approaches utilized a discrepancy method exclusively. The defining feature of such methods is their attempt to quantify the difference between two different measurement tools, with the quantified difference believed to represent camouflaging. A total of 12 used an observation/reflective method. This refers to the use of both questionnaires or direct observation. One study utilized a mixed approach. Eight of the included studies were completed in the United Kingdom; four in the United States, three in Australia and one in Poland.

**TABLE 1:** Publication, name of measure, type of measure, and demographic information for included studies.

Publication	Name of measure	Type of measure	Group	*N*	Age	Sex (male/female/other)	Country of study
Brown et al. (2020)	GQ‐ASC	Observational/reflective	Autistic and non‐autistic cisgender and transgender women	350 autistic 322 non‐autistic	Autistic: 18–71 y/o Non‐autistic: 18–72 y/o	Autistic: 345/4/1 Non‐autistic: 0/322	Australia
Cage and Troxell‐Whitman (2019)	The Cage Questionnaires	Observational/reflective	Autism spectrum, Asperger syndrome, PDD‐NOS	262	>18 y/o *M* = 33.62	111/135/16	United Kingdom
Cassidy et al. (2018)	The Cassidy Questionnaire	Observational/reflective	Autistic and non‐autistic	164 autistic 169 non‐autistic	20–60 Y/O	Autistic: 65/99 Non‐autistic: 54/115	United Kingdom
Cola et al. (2022)	The Cola Method	Observational/reflective	Autistic and non‐autistic	101 autistic 34 non‐autistic	Autistic males *M* = 10.66 Autistic females *M* = 10.15	Autistic: 76/25 Non‐autistic: 20/14	United States
Dean et al. (2017)	The POPE	Observational/reflective	Autistic and non‐autistic	48 autistic 48 non‐autistic	Autistic boys *M* = 7.71 Autistic girls *M* = 7.75 Non‐autistic group *M* = 7.92	Autistic: 24/24 Non‐autistic: 24/24	United States
English et al. (2021)	CATI	Observational/reflective	Autistic and non‐autistic	17 autistic 1149 non‐autistic	18–82 y/o	Autistic: 10/6/1 Non‐autistic: 559/575/15	Australia
Hull et al. (2019)	CAT‐Q	Observational/reflective	Autistic and non‐autistic	354 autistic 478 non‐autistic	Autistic: 16–82 y/o Non‐autistic: 18–75 y/o	Autistic 108/179/67 Non‐autistic: 192/255/31	United Kingdom
Jedrzejewska & Dewey (2022)	CAT‐QO	Observational/reflective	Autistic and non‐autistic	39 autistic 151 non‐autistic	13–19 y/o	Autistic: 26/13 Non‐autistic: 110/41	United Kingdom
Ormond et al. (2018)	The Q‐ASC	Observational/reflective	Parents of autistic children	236	5–19 y/o	138/98	Australia
Parish‐Morris et al. (2017)	The Parish‐Morris Method	Observational/reflective	Autistic and non‐autistic	65 autistic 17 non‐autistic	6–17 y/o Autistic *M =* 9.96 Non‐autistic *M =* 11.32	Autistic: 49/16 Non‐autistic: 8/9	United States
Lai et al. (2017)	The Lai Method	Discrepancy	Autistic	60	18–49 y/o Male *M* = 27.2 Female *M* = 27.8	30/30	United Kingdom
Livingston et al. (2019)	The Livingston Method	Discrepancy	Autistic	136	10–15 y/o *M =* 13.28	112/24	United Kingdom
Livingston et al. (2020)	Compensation checklist	Observational/reflective	Autistic and non‐autistic	58 autistic 59 non‐autistic	18–77 y/o Autistic *M* = 35.83 Non‐autistic *M* = 33.88	Autistic 14/44 Non‐autistic: 8/51	United Kingdom
Rynkiewicz et al. (2016)	The Rynkiewicz method	Observational/reflective	Autistic	26	5–10 y/o	16/10	Poland
Schuck et al. (2019)	The Schuck method	Discrepancy	Autistic	28	Male *M* = 23 Female *M* = 33	17/11	United States
Wood‐Downie et al. (2021)	The Wood‐Downie Method	Mixed	Autistic/high autistic traits and non‐autistic	40 autistic/high autistic traits 44 non‐autistic	Autistic/high autistic traits *M* = 10.08 Non‐autistic *M* = 10.50	Autistic/high autistic traits: 22/18 Non‐autistic: 22/22	United Kingdom

Abbreviations: CATI, Comprehensive Autistic Trait Inventory; CAT‐Q, Camouflaging Autistic Traits Questionnaire; CAT‐QO, Camouflaging Autistic Traits Online Questionnaire; PDD‐NOS, pervasive developmental disorder – not otherwise specified; POPE, Playground Observation of Peer Engagement; Q‐ASC, Questionnaire for Autistic Spectrum Condition; GQ‐ASC, Girls Questionnaire for Autism Spectrum Condition.

Diagnostic status varied across each study. Five were completed exclusively with self‐identified and/or formally diagnosed autistic individuals. Ten were completed with a mixed sample of autistic and neurotypical participants, and one was completed with parents of autistic children.

In terms of age, seven studies were completed exclusively with adults (i.e., equal to, or over, 18 years old). Six were completed exclusively with children. One study was completed with a mix of adults and children, and one with parents.

When considering the sex/gender distribution of the 4455 participants included in the review, 44.5% classified themselves as male, 48.6% identified as female, and 6.9% either identified as neither male or female, or did not wish to disclose. It should be noted that the current paper uses the term sex/gender to recognize that some papers differentiated by sex, whilst others categorized by gender, and many did not define these labels when using them.

#### Quality appraisal

The results of the quality appraisal are presented in Table 2, below. None of the included studies investigated criterion validity or responsiveness, which are part of the COSMIN checklist. For ease of comparison, discrepancy methods and observation/reflective methods will be discussed separately. The results present a summary of each measure's quality appraisal, based on the COSMIN, and identify areas of improvement for each method. Note that scores on individual items of the COSMIN checklist are available upon request from the corresponding author.

**TABLE 2:** COSMIN checklist ratings for all methods.

Name of measure	Internal consistency	Reliability	Measurement error	Content validity	Structural validity	Hypothesis testing	Cross‐cultural validity/measurement invariance	Criterion validity	Responsiveness
Cage et al.	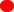	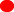	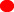	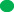	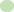	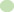	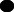	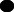	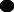
Camouflaging discrepancy (Lai et al. method)	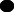	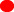	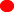	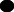	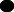	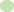	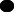	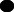	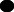
Camouflaging discrepancy (Schuck et al. method)	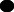	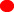	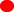	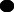	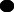	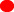	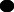	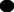	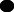
Cassidy et al.	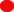	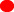	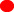	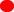	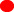	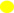	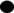	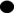	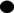
CATI	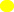	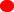	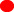	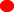	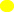	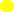	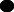	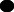	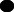
CAT‐Q	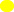	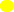	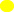	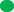	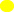	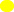	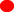	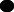	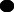
CAT‐QO	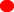	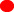	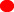	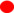	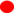	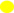	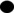	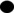	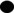
Cola et al.	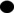	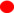	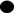	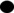	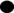	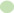	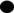	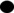	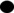
Compensation Checklist	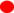	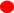	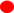	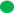	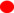	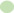	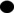	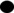	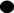
GQ‐ASC	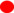	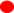	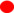	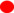	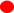	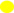	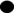	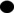	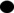
Livingston et al.	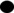	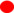	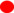	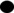	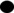	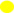	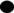	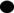	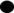
Parish‐Morris et al.	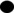	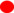	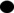	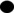	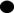	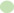	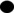	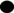	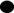
The POPE	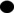	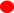	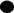	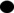	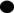	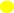	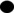	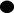	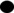
Q‐ASC	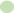	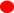	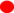	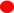	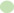	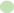	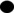	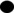	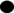
Rynkiewicz et al.	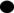	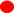	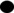	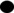	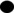	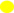	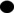	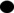	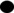
Wood‐Downie et al.	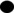	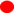	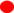	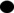	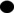	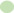	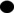	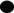	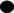

*Note*: Key: 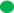 Excellent; 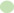 Good; 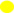 Fair; 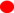 Poor; 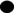 Not assessed.

Abbreviations: CATI, Comprehensive Autistic Trait Inventory; CAT‐Q, Camouflaging Autistic Traits Questionnaire; POPE, Playground Observation of Peer Engagement; Q‐ASC, Questionnaire For Autistic Spectrum Condition; GQ‐ASC, Girls Questionnaire for Autism Spectrum Condition.

#### Discrepancy methods

##### Camouflaging discrepancy (Lai et al., 2017; Lai et al., 2019)

This approach was developed to directly measure camouflaging, defined as a “coping strategy” used by autistic people (Lai et al., 2017). This method first used by Lai et al. (2017) used the Autism Quotient (AQ; Baron‐Cohen, Wheelwright, Skinner, et al., 2001) and the “Reading the Mind in the Eyes” test (RMET) (Baron‐Cohen, Wheelwright, Hill, et al., 2001) as indicators of internal autistic status. External presentation was quantified using the ADOS (Lord et al., 2000). Camouflaging scores were created by mean centering all scores to the sample, and scaling by using the maximum available score. The AQ and RMET scores were subtracted from the ADOS score individually, with the two scores included in a principal component analysis (PCA) to create an overall camouflaging score using the first principal component. It should be noted that the 2017 paper used the Western‐Psychological Services (WPS) published “diagnostic algorithm score”, whilst the 2019 publication used the updated Social Affect domain score (Hus & Lord, 2014). Whilst suggesting two potentially different methods, Lai et al. (2019) repeated their analysis using the original WPS method, with consistent findings.

Psychometrically, the camouflaging discrepancy (CD) method demonstrated relative strengths in terms of its ability to test hypotheses relating to group difference and potential covariates to camouflaging. The continued use of the discrepancy method may now benefit from further understanding the reliability of the camouflaging score across time points, and between raters. It has been suggested that measuring discrepancy through the calculation of discrepancy scores is fundamentally flawed due to the inability to separate the variance of the components of the discrepancy (e.g., ADOS, AQ, RMET), with the discrepancy itself (see, Zuckerman et al., 2002). Instead, using a regression interaction approach, where the interaction between internal and external autistic characteristics can be used to predict the outcome of interest, may be a more optimal approach to exploring CD both within and between groups. This evaluation applies to most existing discrepancy‐based methods, to our knowledge.

##### Livingston et al. (Livingston, Colvert, et al., 2019)

Livingston et al. original method was created to identify and categorize the extent to which autistic individuals compensate for “underlying difficulties” which they stated might partially overlap with camouflaging. The technique quantifies social cognitive ability by using the Frith‐Happé animation task, where higher scores represent greater theory of mind abilities (ToM; Abell et al., 2000), and compares this with external autistic presentation, using the ADOS (Lord et al., 2000), where higher scores represent greater autistic characteristics. This results in a categorical classification of individuals into four pre‐assigned groups, based upon a median split of the data from a typically developing group. Autistic individuals are then classified as being “high compensators” (low ADOS score, low ToM score), “low compensators” (high ADOS, low ToM), “deep compensators” (low ADOS, high ToM), or “unknown” (high ADOS, low ToM).

This method had particular strengths in terms of its ability to test between groups. However, its reliability was limited as these groups were created based on the characteristics of this particular sample (i.e., scoring above/below the sample median for ADOS and ToM). In other samples, the same participants might be categorized differently in relation to a different sample median. Researchers using the method should now evaluate the reliability of group assignment and, if necessary, identify more reliable methods of categorizing participants such as cut‐off scores.

##### Camouflaging discrepancy (Schuck et al., 2019)

The CD method from Schuck et al. (2019) attempted to replicate the findings from Lai et al. (2017) using a North American Sample, thereby directly measuring camouflaging. It should however be noted that this replication did not use the RMET, and no PCA was required. Instead, mean centered data from the ADOS and AQ were used to create a camouflaging score.

The method was successfully used to investigate sex differences with camouflaging, as well as potential covariation between camouflaging, working memory, and emotional expressivity. To improve the method, it would be beneficial to assess whether similar findings could be replicated in a larger sample size. Further information about the reliability and measurement error of the camouflaging score would also help researchers understand the potential utilization of this method in the future.

#### Observational/reflective methods

##### Camouflaging of Autistic Traits Questionnaire (CAT‐Q; Hull et al., 2019)

The CAT‐Q was born out of qualitative research into the nature, motivations, and consequences of camouflaging autistic traits (Hull et al., 2017). It is a direct self‐report measure of camouflaging, using 25‐items, with responses across a seven‐point Likert‐scale. The questionnaire consists of three subscales: compensation (strategies used to compensate for social difficulties), masking (hiding of autistic characteristics or portrayal of neurotypical behavior), and assimilation (attempts to fit into social situations). The CAT‐Q has subsequently been translated into Italian using a student population (Dell'Osso et al. 2022; see Table S1). We note here that two of the authors (W. M. and L. H.) were involved in the original development of the CAT‐Q. Evaluation in this study was performed by the other author (B. H.), however, we acknowledge that this may have influenced our evaluation of the measure.

The questionnaire has several strengths. The questionnaire demonstrates good content validity with its use of prior qualitative research to help inform the creation of the questionnaire. The CAT‐Q has also demonstrated good test–retest reliability in a small subgroup. The CAT‐Q has demonstrated measurement invariance across diagnostic (autistic/non‐autistic) and gender (male/female) groups, and there is emerging evidence for cross‐cultural validity although the factor structure of any translation of the CAT‐Q has not yet been evaluated in peer‐reviewed research. Whilst the questionnaire has reported internal consistency statistics, along with exploratory and confirmatory factor analysis, there is limited information about how missing items should be handled. Considering such factors could help increase the utility of the CAT‐Q outside of research contexts.

##### Camouflaging of Autistic Traits Online Questionnaire (CAT‐QO; Jedrzewska & Dewey, 2022)

The CAT‐QO is an adapted version of the previously mentioned CAT‐Q. It attempts to understand camouflaging behavior using social media platforms. The primary author (L. H.) of the CAT‐Q was consulted during the development of the CAT‐QO. The questionnaire retains the original 25 items and is reworded to reflect a social media environment, for example “I feel that I'm performing rather than being myself” was changed to “On social media I feel that I'm performing rather than being myself.” The questionnaire also retains the seven‐point Likert‐scale for responses.

The strengths of the CAT‐QO appear most evident when attempting to assess hypotheses. This was particularly evident from the authors, who investigated potential group differences between autistic and non‐autistic teenagers. Whilst the questionnaire provides the opportunity to extend camouflaging research into online behavior, it has yet to be tested in terms of its consistency and reliability. It is also unclear as to whether the items from the CAT‐Q adequately reflect camouflaging behavior online. To improve the utility of the questionnaire, it would be beneficial to first investigate whether all the items adequately reflect online camouflaging in autistic teenagers, before further considering statistical constructs such as consistency and reliability.

##### The Comprehensive Autistic Trait Inventory (CATI; English et al., 2021)

The CATI is a relatively new measure that attempts to measure a broad range of autistic traits. The authors describe how previous measures such as the AQ do not adequately incorporate all domains that are associated with autism, such as sensory sensitivities. One such area that the authors argued has not been incorporated into current measurements is social camouflaging, leading to the creation of a seven item social camouflaging subscale within the CATI. Items within this subscale are responded to using a five‐point Likert scale that ranges from “definitely disagree” to “definitely agree.”

The subscale holds several strengths. The authors were able to demonstrate a fair level of internal consistency, which could have been improved by providing information relating to how missing items are handled. The subscale also demonstrated fair structural validity through the use of factor analysis. Further improvements to the subscale could now be gained by understanding constructs such as test–retest reliability, to understand the potential stability or variability in scores over time.

##### The Girls Questionnaire for Autism Spectrum Condition (GQ‐ASC; Brown et al., 2020)

The GQ‐ASC was utilized by Brown et al. to evaluate whether a modified version of this measure could be utilized to screen for autism in adult women. The questionnaire was not originally designed to assess camouflaging, however, after completing PCA, a five‐component solution was extracted; one of which was interpreted as reflecting camouflaging. The subscale is comprised of four questions rated along a four‐point Likert‐scale. Using the scores from this subscale, the authors were able to compare camouflaging between autistic and non‐autistic cisgender and transgender women.

When investigating camouflaging, the GQ‐ASC can measure and test a prior hypotheses about camouflaging behavior in females. However, it should be noted that the primary intention of the questionnaire was not to comprehensively describe camouflaging as a construct. What is unclear, however, is whether these items would be seen as the most relevant and salient indicators of camouflaging by autistic females. In may therefore be beneficial to conduct further research with autistic females to ascertain whether there are omissions from this subscale which could provide a more comprehensive reflection of camouflaging as a construct.

##### Camouflaging reasons and contexts (Cage & Troxell‐Whitman, 2019)

The camouflaging reasons and contexts questionnaires are two separate self‐report measure that do not seek to measure camouflaging directly, but instead investigate the places where camouflaging may occur, and what the reasons are for doing so.

The camouflaging reasons questionnaire has 21 statements requiring agreement or disagreement across a five‐point Likert‐scale. Two principal components were extracted from the questionnaire. The first was labeled “conventional reasons,” where camouflaging serves a primary function in an education or occupational context. The second was named “relational reasons,” where camouflaging aids interpersonal interactions. The questionnaire has been used to investigate potential group differences in camouflaging reasons between males and females.

Similarly, the camouflaging context questionnaire was comprised of 22 common contexts for camouflaging, with respondents indicating how often they camouflaged in that context across a five‐point Likert‐scale. Two components were extracted from this questionnaire, including “formal contexts” (e.g., work/school), and interpersonal contexts (e.g., when with friends or family). The contexts questionnaire has been used to categorize individuals who camouflaged on either a consistently low, consistently high basis, or those that switched between high and low depending upon context. This has enabled between‐group comparisons in levels of anxiety and stress.

The method by which the context and reasons questionnaires were created gives them specific strengths. This was particularly the case for their content validity, given that the questionnaires were created in collaboration with individuals from the autistic community. The questionnaires also demonstrated relative strengths in terms of their structural validity, given the incorporation of exploratory factor analysis. Future research using these questionnaires may now wish to consider their reliability, for example, with the contexts questionnaire, whether participants would remain categorized as consistently high, consistently low, or switchers.

##### Cassidy Questionnaire (Cassidy et al., 2018)

The questionnaire designed by Cassidy et al. (2018) was used to investigate the tendency for someone to camouflage, rather than a thorough representation of camouflaging behaviors, with this score being used to assess a potential association with suicidality in autistic adults. The questionnaire was comprised of four questions, with participants first being asked whether they have tried to mask or hide their autistic symptoms. Those responding yes were then required to report in which contexts this occurs, how often they engage in this behavior, and the overall amount of time per day they spend masking. Scores are calculated as a sum of overall contexts (out of 8), frequency (out of 6), and amount (out of 6). Total camouflaging scores are calculated out of 20.

It should be noted that the questionnaire was not designed with the primary intention of investigating camouflaging. Instead, it was designed to investigate covariation between camouflaging and suicidality. As such, the questionnaire enabled the researchers to investigate this question effectively. Future use of this questionnaire could consider its relationship with other more established measures, and whether participant responses are consistent across time.

##### Cola measure (Cola et al., 2022)

The method used by Cola et al. attempts to measure camouflaging from a linguistic standpoint, by investigating the use of social words. This was completed by analyzing the interview section from module three of the ADOS. The authors then measured the number of social category words, friend category words, and family category words by each child. When investigating differences between the sexes, the authors found that autistic girls used more social words than autistic boys. Social word use was also correlated with ADOS‐2 SA scores, suggesting that the use of social words led to lower ratings of impairment by the assessing clinician. Such results were interpreted as supporting the idea that camouflaging behavior may be supported by the use of social language.

The Cola method provides researchers with the ability to measure a potential indicator of camouflaging by using an interview‐based approach that will be familiar to many clinicians, producing objective, quantifiable data. This has led to clear strengths in the measures ability to test hypotheses, such as sex differences in social language use.

However it is not clear to what extent this method measures camouflaging as a construct, as opposed to specific linguistic examples of camouflaging; the measure's reliability and construct validity require further examination.

##### The Playground Observation of Peer Engagement (POPE; Dean et al., 2017)

The POPE is a measurement tool that investigates playground interactions in children. It has been used to classify behavior into pre‐assigned categories (Kasari et al., 2011; Kasari et al., 2016). For the purposes of measuring camouflaging, three observation categories are used: game (child is actively playing a game with another), joint engagement (child is socializing with others), and solitary (child is alone). Each child is classified as being within of these three states every 1 min, across a 10‐ to 15‐min observation period. The POPE has been used to compare autistic and non‐autistic children across sex and diagnostic status. Dean et al. (2017) observed frequent weaving in and out of joint engagement for autistic girls, which the authors suggested was a representation of camouflaging; in other words, this method was not developed to measure camouflaging directly.

The POPE is a novel measure that enables children to be observed within a natural setting. Such information can be rich, and is free from experimental manipulation. Through Dean et al.'s adjustment to the tool for the measurement of camouflaging, it possible to understand the behavioral differences of autistic girls in the playground, compared to boys. The authors also demonstrated that using such a tool can provide good levels of inter‐rater reliability. Users of the tool may now wish to examine its test–retest reliability to understand whether factors such as the presence of other children in the playground would lead to changes in behavior from the child being observed.

##### The Questionnaire for Autism Spectrum Conditions (Q‐ASC; Ormond et al., 2018)

The Q‐ASC is a questionnaire not specifically developed to measure camouflaging, but instead to measure broader autism symptomology. It is completed by parents of five to 19‐year‐olds, and has a specific subscale relating to masking of emotions and responses during social interactions. The subscale is comprised of five questions rated along a four‐point Likert‐Scale. Using the scores from this subscale, the authors were able to compare social masking between males and females.

The social masking subscale of the Q‐ASC holds particular psychometric strengths. Specifically, the subscale holds good structural validity, given that its creation was based upon exploratory factor analysis of a pre‐existing questionnaire. Given such methodology, the subscale also demonstrates good levels of internal consistency. It should however be noted that the social masking subscale was designed to measure masking of emotional responses and expressions, rather than the broader definitions of masking/camouflaging described in other literature. As such, it may be beneficial to now triangulate the outcomes of the statistical analysis with the experiences of those from the autistic community, prior to future investigations of reliability.

##### Parish‐Morris measure (Parish‐Morris et al., 2017)

The method used by Parish‐Morris et al. focuses specifically upon linguistic strategies that may contribute towards successful social camouflaging. As such, it is not a direct measure of social camouflaging per se. The technique measures filled pauses, differentiating between “UH”, which is used to signal a short delay, and “UM”, which is used to signal more significant delays. For autistic children, lower use of “UM” is associated with autistic symptomology. When investigating differences between sexes and those with and without a diagnosis, Parish‐Morris et al. (2017) found that autistic girls, and typically developing children having higher “UM” ratios when compared to autistic boys, during an ADOS. The authors proposed that this difference represents linguistic camouflaging, providing females with the opportunity to blend in with their typically developing peers.

The ability to investigate linguistic strategies that contribute towards potential camouflaging provides a novel way to quantify specific techniques that may or may not be conscious to the individual. Such quantifiable data enables individuals to then test a priori hypotheses but could now benefit from further investigations into the reliability of such strategies across both time and contexts.

##### Compensation checklist (Livingston et al., 2020)

The compensation checklist is designed to measure compensation strategies in autism, as previously defined (Livingston et al., 2019), rather than camouflaging directly. The checklist comprises 31‐items that are scored following a qualitative free text description of compensatory strategies used in social situations, creating a quantitative compensation score. The overall score is comprised of four distinct strategy types: masking (6 items), shallow compensation (10 items), deep compensation (9 items) and accommodation (6 items). The checklist was used to investigate covariation in scores between those with and without a diagnosis of autism, autistic traits, education level, sex, and age of diagnosis.

Whilst the checklist was developed and tested as a way of quantitatively coding compensation in data from a qualitative study, the authors envision the instrument being developed for somewhat different use in future. This would be as a set of prompts for a clinical interview about compensatory strategies; and as the basis for development of a questionnaire. Content validity of the checklist is a particular strength, given that it was created alongside individuals from the autistic community. The ability to create quantitative scores from free‐text responses also enables the users of this checklist to complete hypothesis testing. Use of the checklist, especially if it is developed into a clinical interview, would benefit from further information relating to inter‐rater reliability, and whether training may be required to achieve acceptable levels of reliability. Further research to understand the internal consistency of each individual strategy type would also be beneficial. Although evidence for internal reliability was presented, the factor structure of the Compensation Checklist has not been explicitly tested.

##### Rynkiewicz measure (Rynkiewicz et al., 2016)

The method used by Rynkiewicz et al. investigated non‐verbal behaviors that may facilitate successful social camouflaging. It was developed with five to 10‐year‐old children during the ADOS demonstration task. The child's head, neck, shoulder, elbow, wrist, palm, and finger movements are tracked using a Microsoft Kinect sensor system. This enables a comparison between groups for vividness of gestures (i.e., shorter time of gesture, but increased length). The authors suggest that increased vividness camouflages other autistic diagnostic features; no direct measure of camouflaging itself is produced through this measure.

The use of such modern technology provides a unique way to investigate camouflaging in autistic individuals. Such a technique may be particularly valuable in younger children who may have difficulty verbalizing their camouflaging strategies and enables researchers to quantify and compare behavior. Future evaluation of this method may now wish to investigate the measure's reliability across test contexts.

##### Wood‐Downie measure (Wood‐Downie et al., 2021)

The research from Wood‐Downie et al. (2021) is the only research paper to incorporate a mixed methods approach to the operationalization of camouflaging, both of which use previously developed tools as a proxy for camouflaging. They first utilized an observational/reflective method to investigate “behavioral camouflaging.” This was derived from the interactive drawing test (IDT, van Ommeren et al., 2012, 2017), which investigates social reciprocity. The authors also used the RMET‐C scores (Baron‐Cohen et al., 2001) to operationalize compensatory camouflaging. The results from this study indicated that girls with autism/high‐autistic traits had higher levels of social reciprocity than boys with autism/high‐autistic traits, but that they both had similar social cognitive abilities, which were interpreted as a reflection of increased camouflaging in females.

The mixed methods used by Wood‐Downie et al. (2021) to draw conclusions about sex differences in camouflaging provide another approach for investigating camouflaging between groups. The authors provided clear and testable hypotheses. The approach has clear face validity, given the authors' findings. It should however be noted that, much like the original methods used by Lai et al. (2017, 2019), the tools being used are not specifically designed to investigate camouflaging. As such, the individual results of each child are difficult to interpret. Future research should consider using more rigorous statistical analyses when comparing group differences on CD measures. For instance, looking at both main and interaction effects of the component parts of a discrepancy measure (in this example, social cognition and emotion recognition) to better elucidate whether group differences arise from the individual measures or from differences in discrepancy.

### Discussion

This review identified 16 distinct camouflaging measures. None of the papers included in the review were published prior to 2016. It is therefore unsurprising that many of the available measures are in the preliminary phases of development, with uncertain psychometric properties.

The discrepancy methods identified here demonstrated strengths in terms of their content validity, since they map well onto the concept of camouflaging, by seeking to contrast a person's internal autistic state (“how autistic they really are”) with observations (“how autistic they appear”). However, it should also be noted that they suffer from a conceptual and practical limitation; that in the absence of any diagnostic biomarkers, it is not possible to directly measure a person's internal autistic state independently of how they behave or the experiences they report (Fombonne, 2020). To understand how serious a limitation this is, empirical work is needed to investigate the reliability and validity of discrepancy measures. Most previous studies using the discrepancy approach have not taken into account collinearity between the contributing variables, using zero‐order correlations rather than partial correlations. This is a common criticism of discrepancy measures in other fields, when combining two tools measuring different constructs (in this case, internal and external autistic traits), to estimate another, novel, construct (Zuckerman et al., 2002). Future research should address this by calculating partial correlations between discrepancy measures and theoretically related constructs, which take into account the component variables. One way to do this would be to measure discrepancy via the interaction between variables measuring internal and external autism status, rather than by calculating difference scores. This would allow for better estimation of criterion validity by comparing the discrepancy approach to other methods of measuring camouflaging.

Based on our literature review, none of the discrepancy measures have yet been evaluated in terms of their test–retest reliability. Furthermore, data on validity are also lacking. For example, whilst evidence of higher scores (greater discrepancy) by women and girls compared to men and boys is consistent with a measure being a true index of camouflaging (e.g., Lai et al., 2017) this can only, on its own, be considered weak and preliminary evidence for validity. It is possible that higher scores by females could actually be indexing another inter‐sex/gender difference in autistic presentation (Bargiela et al., 2016). A step towards gaining more evidence on validity would be to simultaneously give participants different measures of camouflaging, including discrepancy and reflective/observational approaches, and to evaluate their associations. Such studies are currently lacking.

Similarly to the discrepancy measures, evidence of content validity was demonstrated for several observation/reflective methods. For example, questionnaires were either shaped by consultation with autistic people (e.g., Camouflaging Reasons and Contexts Questionnaires) or based on careful analysis of reports by autistic people on their experiences of camouflaging (e.g., CAT‐Q, Compensation Checklist). But, as for discrepancy methods, there is limited empirical data on the psychometric properties of observational/reflective methods. Only one, the CAT‐Q, currently has any estimate of test–retest reliability (Hull et al., 2019), and validity data are also lacking. As described above, studies examining the correlations between observational/reflective methods and discrepancy methods will be a useful step towards better understanding of their validity. For instance, discriminant validity could be tested by comparing both discrepancy and observational/reflective methods to measures of theoretically related, but distinct, constructs, such as autistic traits or social communication abilities.

The review highlights some of the key strengths and weaknesses of the available camouflaging measures when assessed by the COSMIN. There were however areas that were not assessable and may be important for future research. The available research was mainly completed within anglophone, industrialized nations (the United Kingdom, United States, Australia). Given that camouflaging can be thought of as arising due to a complex interaction between the autistic individual and their environment (Cage & Troxell‐Whitman, 2019), it is likely that camouflaging will take different forms across cultures. Future camouflaging research should consider this and make adjustments to the measurement tools as necessary. Similarly, the studies included here used a variety of different samples to develop and test methodologies, including formally diagnosed and self‐diagnosed autistic people and those with high levels of autistic characteristics. It may be that types or behavioral impacts of camouflaging differ across these groups, perhaps reflecting different clinical or diagnostic outcomes. Future research should systematically compare methods across all these samples to better understand which methods are most appropriate for which groups, and to determine whether methods can be used with both autistic and neurotypical populations.

None of the available methodologies investigated responsiveness of their measure; that is, changing camouflaging scores in response to true changes in camouflaging behavior. Whilst this would be advantageous, it should be acknowledged that many of the camouflaging measures placed a different emphasis on when camouflaging takes place. For example, observational methods were concerned with potential camouflaging within an allotted time, in front of the examiner. Comparatively, questionnaires consider the historical nature of, attitudes towards, and intentions of camouflaging. As such, many of the questionnaires may require changes and revalidation to specify time periods of interest to measure responsiveness. Further, it is notable that our literature review found only one parent‐report measure of camouflaging, which was designed to address only a limited range of behaviors in the context of measuring autistic characteristics more broadly. The lack of such measures constrains our ability to study camouflaging in young people, and also limits clinical assessment in child and adolescent services.

## STUDY 2: COMPARING DIFFERENT WAYS OF MEASURING CAMOUFLAGING

The results of Study 1, the systematic review, suggest that empirical studies are needed to assess the validity of different approaches to measuring camouflaging. One potentially fruitful approach is to simultaneously give different measures to participants, and to examine their associations with each other. Further, the literature review identified limited parent‐report measures of camouflaging. Study 2 sought to begin to fill these gaps in the literature, by comparing two previously established methods of measurement (self‐report and discrepancy) with a novel, parent‐report method. The aim of this study was to examine concurrent validity of camouflaging measures, by comparing multiple different ways of measuring camouflaging in an adolescent population. A secondary aim was to examine discriminant validity by comparing these methods of measuring camouflaging with a measure of social skills.

### Methods

#### Participants

Previous measure validation work has found moderate‐to‐large correlations between self‐report questionnaires completed by autistic children and their parents (Strang et al., 2017). Using these anticipated effect sizes, a priori power analysis determined that a sample size of 37 would be necessary to detect large, one‐tailed correlations (*r* ≥ 0.40) with power = 0.80 between two measures of camouflaging. A total of 59 participants aged 13–18 (29 female, 30 male) were recruited from healthcare services and through social media and word of mouth from across the United Kingdom. This sample has been previously reported upon in Hull et al. (2020b).

#### Measures

##### Autism Diagnostic Observation Schedule, Version 2 (ADOS‐2; Lord et al., 2012)

A gold‐standard semi‐structured behavioral assessment tool for autistic characteristics. Modules 3 and 4 (aimed at verbally fluent adolescents and adults) were used for all participants in this study. A standardized Calibrated Severity Score (CSS) was calculated for each participant based on the most up‐to‐date algorithms (Hus & Lord, 2014; Lord et al., 2012), with cut‐off scores of 4 or above indicating clinically significant characteristics associated with an autism spectrum disorder.

##### Autism quotient (Baron‐Cohen, Wheelwright, Skinner, et al., 2001)

A standardized self‐report measure of autistic traits comprised of 50 items, suitable for clinical and subclinical populations. The AQ has demonstrated acceptable test–retest reliability and inter‐rater reliability (Baron‐Cohen, Wheelwright, Skinner, et al., 2001). Scores can range from 0 to 50; a score of 32 or above indicates clinically significant levels of autistic traits. Internal consistency in the current sample was acceptable (*α* = 0.85).

##### Camouflaging Autistic Traits Questionnaire (Hull et al., 2018)

This is a 25 item self‐report measure of camouflaging strategies. The CAT‐Q has demonstrated good test–retest reliability and measurement invariance in autistic and non‐autistic adults, and has been used previously with adolescents (Jorgenson et al., 2020). Scores can range from 25 to 175. Internal consistency in the current sample was good (*α* = 0.91).

##### Camouflaging Autistic Traits Questionnaire: Parent‐report

This is an adaptation of the CAT‐Q, with phrasing changed from “I…” to “My child…” for each item; no other changes were made to the original wording or structure. Scores can range from 25 to 175. Internal consistency in the current sample was good (*α* = 0.90). See Supporting Information.

##### Social Responsiveness Scale, Edition 2 (SRS‐2; Constantino & Gruber, 2012)

A standardized parent‐report measure of a child's social abilities, comprised of 65 items. Acceptable levels of reliability and validity have been found in a general population sample of British children (Wigham et al., 2012). A standardized T‐score is calculated for each child, with a mean of 50 and standard deviation of 10. Total scores of 60 and above are indicative of clinically significant social difficulties associated with autism. The Social Communication Impairment subscale (SCI; comprised of 53 items) was used as a proxy of social difficulties in these analyses, with higher scores representing greater social difficulty.

##### Wechsler Abbreviated Scale of Intelligence, Second Edition (WASI‐II; Wechsler, 2011)

The WASI‐II is a standardized measure of intellectual ability suitable for children and adults aged 6–90 years. The WASI‐II has demonstrated good‐to‐excellent internal consistency in both child and adult populations (McCrimmon & Smith, 2012). Full‐scale IQ scores were calculated for each participant in the current analyses.

#### Procedure

Assessments took place at participants' home or school, or at private testing rooms at a university. All measures were administered by doctoral students, who were trained to research‐level reliability by an experienced ADOS trainer. Written informed consent was obtained from parents of all adolescents, and all adolescents also gave their written and verbal assent before data collection began.

Adolescents completed the ADOS Module 4 (unless they had completed an ADOS Module 3 or 4 within the previous 12 months at their local clinic, and the family had given consent for this to be accessed), which was video recorded, in a private room. Adolescents then completed the WASI‐II (unless a WASI or WISC‐IV measure of IQ had already been recorded in the participant's medical notes, and the family had given consent for this to be accessed), and the self‐report CAT‐Q and AQ. Parents completed the parent‐report CAT‐Q and SRS.

#### Statistical analyses

All analyses were performed in R (R Core Team, 2013). Some participants had missing data: 3 participants (5%) were missing IQ scores, 5 (8%) were missing ADOS scores, 7 (12%) had incomplete AQ data, 3 (5%) had incomplete self‐reported CAT‐Q data, 4 (7%) had incomplete parent‐reported CAT‐Q data, and 5 (8%) had incomplete SRS‐2 Social Communication Impairment subscale data. Multiple imputation (using the Hmisc function in R) was used to replace data using plausible values, with all variables used to calculate missing values, and 5 imputed datasets pooled to produce estimates.

Following Schuck et al. (2019), CD scores were calculated to produce a discrepancy score between internal autistic characteristics (AQ) and external presentation (ADOS) for each participant. ADOS CSS and AQ scores were mean‐centered to the whole sample (*N* = 59) and scaled (divided by the maximum possible score of each), and CD scores were calculated as standardized AQ score minus standardized ADOS score.

A correlation matrix (Table 3) was used to examine relationships between self‐reported and parent‐report camouflaging total score and subscales, CD, and parent‐reported social difficulties. Mean scores on all variables are reported in Table 4. Scores on total self‐and parent‐reported CAT‐Q were not significantly different (*t*[58] = −0.18, mean difference = −2.19, 95% CI = −9.44 to 7.85).

**TABLE 3:** Correlations between measures of camouflaging and social skills (*N* = 59).

	SR CAT‐Q Total	SR CAT‐Q compensation	SR CAT‐Q masking	SR CAT‐Q assimilation	PR CAT‐Q Total	PR CAT‐Q compensation	PR CAT‐Q masking	PR CAT‐Q assimilation	Camouflaging discrepancy	SRS‐2 SCI	ADOS CSS
SR CAT‐Q Compensation	0.89***										
SR CAT‐Q Masking	0.69***	0.49***									
SR CAT‐Q Assimilation	0.72***	0.48***	0.19								
PR CAT‐Q Total	0.51***	0.37**	0.51***	0.33*							
PR CAT‐Q Compensation	0.40**	0.30*	0.42***	0.22	0.86***						
PR CAT‐Q Masking	0.37**	0.27*	0.46***	0.16	0.80***	0.51***					
PR CAT‐Q Assimilation	0.45***	0.33*	0.29*	0.45***	0.69***	0.48***	0.28*				
Camouflaging Discrepancy		0.17	0.24	0.23	0.48***	0.45***	0.35**	0.32*			
SRS‐2 SCI	−0.04	−0.06	−0.12	0.10	0.02	0.04	−0.35**	0.48***	0.12		
ADOS CSS	−0.12	−0.05	−0.31*	0.04	−0.49***	−0.47***	−0.45***	−0.20	−0.89***	0.01	
AQ Total	0.33*	0.26*	−0.11	0.58***	0.03	0.03	−0.17	0.28*	0.35	0.28*	0.12

Abbreviations: ADOS CSS, Autism Diagnostic Observation Schedule, Calibrated Severity Score; AQ, Autism Quotient; CAT‐Q, Camouflaging Autistic Traits Questionnaire; PR, parent report; SR, self‐report; SRS‐2 SCI, Social Responsiveness Scale, 2nd Edition, Social Communication Impairment.

* *p* < 0.05.

** *p* < 0.01.

*** *p* < 0.001.

**TABLE 4:** Mean scores on all variables (*N* = 59).

Variable (range)	Mean (*SD*)
Age (13–18)	14.47 (1.74)
IQ (65–130)	100.56 (15.78)
Self‐report CAT‐Q (62–169)	104.54 (25.49)
Self‐report CAT‐Q compensation subscale (16–61)	36.39 (12.86)
Self‐report CAT‐Q masking subscale (12–56)	35.98 (9.27)
Self‐report CAT‐Q assimilation subscale (11–53)	33.56 (10.25)
Parent‐report CAT‐Q (56–164)	106.73 (22.12)
Parent‐report CAT‐Q compensation subscale (17–60)	38.12 (10.15)
Parent‐report CAT‐Q masking subscale (8–52)	27.95 (10.31)
Parent‐report CAT‐Q assimilation subscale (21–56)	40.66 (7.50)
ADOS CSS (0–10)	5.93 (2.82)
Autism quotient (7–45)	25.53 (8.32)
Camouflaging discrepancy (N/A)	0.33 (0.30)
SRS‐2 SCI (47–90)	78.98 (9.80)

Abbreviations: CAT‐Q, Camouflaging Autistic Traits Questionnaire; SRS‐2 SCI, Social Responsiveness Scale, 2nd Edition, Social Communication Impairment.

### Results

Total self‐reported and parent‐report CAT‐Q scores were found to be strongly positively correlated (*r* = 0.51, *p* < 0.001). Total self‐report CAT‐Q scores were weakly positively correlated with CD, although the association was minimally significant (*r* = 0.27, *p =* 0.042). In contrast, total parent‐report CAT‐Q scores were strongly positively correlated with CD (*r* = 0.486, *p* < 0.001). Neither self‐report nor parent‐report total scores were significantly correlated with social communication difficulties (see Table 3). Interestingly, the parent‐reported masking subscale was moderately negatively correlated with social communication difficulties (*r* = −0.35, *p =* 0.007), while parent‐reported assimilation was strongly positively correlated with social communication difficulties (*r* = 0.48, *p* < 0.001).

### Discussion

Good internal consistency was found for both self‐report (*α* = 0.91) and parent‐report (*α* = 0.90) CAT‐Q. A strong positive correlation was found between self‐report and parent‐report total CAT‐Q scores, suggesting that adolescents and their parents have relatively similar perceptions of the adolescent's camouflaging strategies. This suggests that the parent‐report CAT‐Q could be used in cases where young people are unable to report their own camouflaging strategies, although discrepancies between raters are still observed. However, it should be noted that the correlation between these two measures was not high enough to suggest full convergent validity (Carlson & Herdman, 2010), and therefore the measures should not be considered fully interchangeable. Moderate correlations between the self‐report and parent‐report CAT‐Q subscales of compensation, masking, and assimilation were also observed, suggesting that parents and young people may pick up on different behaviors related to each of these constructs as well as identifying some similar behaviors.

The self‐ and parent‐report CAT‐Q scores were compared to a previously established discrepancy method of measuring camouflaging; the Camouflaging Discrepancy (CD) score. Total self‐reported camouflaging was only weakly correlated with CD, and none of the self‐report subscales were significantly correlated with CD. In contrast, parent‐reported camouflaging total and subscale scores were all moderately and significantly correlated with CD. CD scores represent the observable consequences of camouflaging strategies; individuals who have higher CD scores demonstrate fewer autistic behaviors during observation (using the ADOS), than would be predicted by their self‐reported level of autistic traits (Lai et al., 2017).

The parent‐report CAT‐Q may also be sensitive to the external impact of camouflaging strategies, as parents can only report on strategies that they observe their child using or are reported to them by the child. This may account for the moderate correlation between parent‐report CAT‐Q and CD. Conversely, the self‐report CAT‐Q measures the individual's own perception of their use of strategies, regardless of impact on behavior (Hull et al., 2018). Adolescents may have self‐identified camouflaging strategies which required more effort but did not produce as many behavioral changes, resulting in a weaker association between self‐report CAT‐Q and CD. The correlations between all three of these measures were only moderate at their highest, however, suggesting that the three approaches may each identify different facets or features of camouflaging.

We would like to note again here the previously discussed limitations of calculating discrepancy scores. While the parent‐report CAT‐Q appears to correlate moderately with established methods of measuring CD, we repeat the recommendations of others to measure CD through a regression interaction method. Once this method has been tested within an appropriately sized sample, we strongly recommend comparison of the self‐ and parent‐report CAT‐Q and a discrepancy interaction method, to better test the convergent validity of camouflaging measures. This would allow for more accurate comparisons between methods of measuring camouflaging, and therefore better understanding of the construct(s) being measured by these methods.

Neither self‐ nor parent‐report total camouflaging measure, nor the CD score, was significantly correlated with social difficulties, as measured by the Social Communication Impairment subscale of the SRS‐2. This suggests that the overall construct of camouflaging (however measured) is distinct from an individual's social abilities, although parents may consider their child's social skills when reporting on the assimilation components of camouflaging. In other words, camouflaging is not the same as simply having relatively good social skills (based on neurotypical social expectations), and the need to camouflage may be driven by other factors rather than poor social skills alone, such as the experience of stigma (Pearson & Rose, 2021; Perry et al., 2021).

Strengths of this study include a balanced sample of male and female adolescents, reducing the potential for sex/gender bias in assessing camouflaging. The self‐report CAT‐Q allows individuals to directly report on their own camouflaging experiences, and was developed from autistic adults' descriptions of camouflaging (Hull et al., 2017), but is limited in its use to individuals who are cognitively and verbally able to do so. The parent‐report CAT‐Q overcomes this issue to some extent, as it can be completed on behalf of others, but it has not been psychometrically validated yet, and so its factor structure and reliability are still untested. There is no research to our knowledge about camouflaging in autistic individuals with no or minimal spoken language, therefore we do not know whether the parent‐report CAT‐Q measures camouflaging behaviors that would be expressed by these populations. The self‐report CAT‐Q has also not been validated for use with adolescents. However, the results of this study and others (e.g., Jorgenson et al., 2020), suggest that it is feasible to use the self‐report CAT‐Q with verbally and intellectually able autistic teenagers.

Although this study explored the discriminant validity of the CAT‐Q by comparing with social skills, the present analysis did not address the potential overlap between the CAT‐Q and measures of social anxiety (Fombonne, 2020). We recommend future research evaluate the discriminant validity between the CAT‐Q subscales and/or items and social anxiety, to better understand how camouflaging is similar and/or distinct from social anxiety. We also suggest that future research using larger sample sizes explores the item‐level similarities and differences between the self‐report and parent‐report CAT‐Q, to better understand how individual camouflaging behaviors might be captured. Item‐level analyses would identify which camouflaging behaviors are picked up by both parents and young people, and which may be reported by one rater only.

Finally, research using discrepancy measures of camouflaging, including this study, has been limited in its ability to control for additional factors that may impact discrepancy scores, such as group differences on the proxy measures of autistic characteristics used to produce a discrepancy score. This could be addressed in two ways: (1) by developing more accurate methods of measuring autistic characteristics, which will be less influenced by confounders, and (2) by controlling for the component parts of discrepancies scores when performing group comparisons, so that any variance is attributed to discrepancy only.

## GENERAL DISCUSSION

Triangulation of measurement, using multiple different methods, is important to achieve a more precise understanding of the true nature of camouflaging. However, individual methods should be used where they are the most appropriate. For instance, self‐report would be most helpful when assessing the impact of camouflaging on mental health or wellbeing in verbally able adults or adolescents, as the individual will be able to describe strategies which may not affect their behavioral presentation, but may require extensive effort. If the individual is less able to report on their own behaviors, parent‐report can be used to provide an estimate of behaviors which may not be observable during a short clinical or research assessment. Researchers might wish to use other observational methods to explore camouflaging in greater depth, including its impact on peer relations. If a researcher wishes to identify both the autistic traits being masked or compensated for, and the impact that this has on an individual's behavioral presentation, a discrepancy approach is most appropriate to quantify camouflaging which the individual may not even be aware of.

These two studies evaluated existing methods of measuring camouflaging in autism, and directly compared three methods in an autistic adolescent sample. All the methods identified here demonstrated strengths and weaknesses, suggesting that no current method fully meets all evaluation criteria. However, the empirical comparison provided preliminary evidence that self‐report, parent‐report, and discrepancy methods all measure a similar underlying construct of camouflaging. While existing methods are better evaluated, and future methods are developed, we suggest choosing from existing methods based on the specific research or clinical need in each case.

These studies have demonstrated that camouflaging is likely comprised of multiple related concepts which may vary between and within individuals. This is reflected in the variety of approaches to measuring camouflaging, such as self‐report and discrepancy, which may overlap to some extent but also capture different components of camouflaging. One discrepancy approach was tested empirically here, but it remains to be seen whether discrepancy methods using alternate proxies for internal and external autistic characteristics demonstrate similar independence from self‐ and parent‐report methods.

It is also important to note that these different methodologies may also be differently impacted by confounding factors or additional variables. For instance, discrepancy approaches may be more strongly influenced by measurement error, but may allow for better consideration of clinical outcomes. In contrast, observational/reflective methods may be more influenced by individual characteristics such as self‐awareness, but may better capture internal aspects of camouflaging.

Novel methodologies have been proposed which may better characterize specific components of camouflaging, such as neuroimaging to identify unconscious or innate camouflaging behaviors (Lai et al., 2021), as well as computational models which allow for more precise testing of the relationships between different components of camouflaging (Ai et al., 2022). It is imperative that future research directly compares both approaches to measuring camouflaging described here, in addition to methods developed in the future. This will allow identification of the areas in which camouflaging measures can be considered overlapping, as well as the extent to which camouflaging as a construct might be broken down into distinct components.

## CONFLICT OF INTEREST

Laura Hull and Will Mandy were part of the original research team developing the CAT‐Q, which is evaluated in the review; however, they receive no financial gains from this freely available resource. The authors have no other conflicts of interest to report. There has been no financial gain during the research. Ben Hannon produced the systematic review as part of his DClinPsy which was funded by Health Education England. Will Mandy's work is funded by the National Institute for Health Research, Autistica, the Medical Research Council, the European Research Council and Dunhill Medical Trust. Laura Hull is currently supported by the Elizabeth Blackwell Institute, University of Bristol, the Wellcome Trust and the Rosetrees Trust, but was not at the time this research was conducted.

## ETHICS STATEMENT

Ethical approval for this study was obtained from the Health Research Authority and the Bloomsbury Research Ethics Committee (Reference 17/LO/2055).

## Supporting information


**Appendix S1** Supporting Information.

**Table S1** Additional papers identified during the literature search.

## Data Availability

The data that support the findings of this study are available from the corresponding author upon reasonable request.
