# Supplementary material for: A comparison of methods for measuring camouflaging in autism
Source: Autism Res. 2022 Nov 24;16(1):12–29. doi: 10.1002/aur.2850 (PMC10099783; doi:10.1002/aur.2850)
Supplement: Supplementary file 1 — Appendix S1 Supporting Information. [file AUR-16-12-s002.pdf]

Supplemental information one – COSMIN Checklist with 4-Point Scale

Measurement property	Definition	Number of measurement items
Internal consistency	The extent to which items on the measurement tool are interrelated	11
Reliability	The degree to which scores from participants who have not changed are the same under repeated measurements.	14
Measurement error	The amount of random error that is not due to changes in the variable of interest	11
Content validity	The extent to which the measurement instrument is measuring what it reports to measure	5
Structural validity	How much the measurement tool reflects the dimensionality of the construct under measurement	7
Hypothesis testing	The extent to which the measurement tool responds as expected under hypothesis testing conditions	10
Cross cultural validity	The degree to which the measurement tool adequately reflects the original measurement tool, after translation	15
Criterion validity	The degree to which the measurement tool performs with a pre-set criterion	7
Responsiveness	The ability for a measurement tool to detect change, in the context of true changes in the construct of interest	18

COSMIN checklist with 4-point scale

Contact
CB Terwee, PhD
VU University Medical Center
Department of Epidemiology and Biostatistics
EMGO Institute for Health and Care Research
1081 BT Amsterdam
The Netherlands
Website: www.cosmin.nl, www.emgo.nl
E-mail: cb.terwee@vumc.nl

Instructions

This version of the COSMIN checklist is recommended for use in systematic reviews of measurement properties. With this version it is possible to calculate overall methodological quality scores per study on a measurement property. A methodological quality score per box is obtained by taking the lowest rating of any item in a box ('worse score counts'). For example, if for a reliability study one item in the box 'Reliability' is scored poor, the methodological quality of that reliability study is rated as poor. The Interpretability box and the Generalizability box are mainly used as data extraction forms. We recommend to use the Interpretability box to extract all information on the interpretability issues described in this box (e.g. norm scores, floor-ceiling effects, minimal important change) of the instruments under study from the included articles. Similar, we recommend to use the Generalizability box to extract data on the characteristics of the study population and sampling procedure. Therefore no scoring system was developed for these boxes.

This scoring system is described in this paper:

Terwee CB, Mokkink LB, Knol DL, Ostelo RWJG, Bouter LM, de Vet HCW. Rating the methodological quality in systematic reviews of studies on measurement properties: a scoring system for the COSMIN checklist. *Quality of Life Research* 2012.[464]

Step 1. Evaluated measurement properties in the article

	Internal consistency	Box A
	Reliability	Box B
	Measurement error	Box C
	Content validity	Box D
	Structural validity	Box E
	Hypotheses testing	Box F
	Cross-cultural validity	Box G
	Criterion validity	Box H
	Responsiveness	Box I

Step 2. Determining if the statistical method used in the article are based on CTT or IRT

Box General requirements for studies that applied Item Response Theory (IRT) models		excellent	good	fair	poor
1	Was the IRT model used adequately described? e.g. One Parameter Logistic Model (OPLM), Partial Credit Model (PCM), Graded Response Model (GRM)	IRT model adequately described	IRT model not adequately described		
2	Was the computer software package used adequately described? e.g. RUMM2020, WINSTEPS, OPLM, MULTILOG, PARSCALE, BILOG, NLMIXED	Software package adequately described	Software package not adequately described		
3	Was the method of estimation used adequately described? e.g. conditional maximum likelihood (CML), marginal maximum likelihood (MML)	Method of estimation adequately described	Method of estimation not adequately described		
4	Were the assumptions for estimating parameters of the IRT model checked? e.g. unidimensionality, local independence, and item fit (e.g. differential item functioning (DIF))	assumptions of the IRT model checked	assumptions of the IRT model partly checked	assumptions of the IRT model not checked or unknown	

To obtain a total score for the methodological quality of studies that use IRT methods, the 'worse score counts' algorithm should be applied to the IRT box in combination with the box of the measurement property that was evaluated in the IRT study. For example, if IRT methods are used to study internal consistency and item 4 in the IRT box is scored fair, while the items in the internal consistency box (box A) are all scored as good or excellent, the methodological quality score for internal consistency will be fair. However, if any of the items in box A is scored poor, the methodological quality score for internal consistency will be poor.

Step 3. Determining if a study meets the standards for good methodological quality

Box A. Internal consistency		excellent	good	fair	poor
1	Does the scale consist of effect indicators, i.e. is it based on a reflective model?				
Design requirements					
2	Was the percentage of missing items given?	Percentage of missing items described	Percentage of missing items NOT described		
3	Was there a description of how missing items were handled?	Described how missing items were handled	Not described but it can be deduced how missing items were handled	Not clear how missing items were handled	
4	Was the sample size included in the internal consistency analysis adequate?	Adequate sample size (≥100)	Good sample size (50-99)	Moderate sample size (30-49)	Small sample size (<30)
5	Was the unidimensionality of the scale checked? i.e. was factor analysis or IRT model applied?	Factor analysis performed in the study population	Authors refer to another study in which factor analysis was performed in a similar study population	Authors refer to another study in which factor analysis was performed, but not in a similar study population	Factor analysis NOT performed and no reference to another study
6	Was the sample size included in the unidimensionality analysis adequate?	7* #items and ≥100	5* #items and ≥100 OR 6-7* #items but <100	5* #items but <100	<5* #items

		excellent	good	fair	poor
7	Was an internal consistency statistic calculated for each (unidimensional) (sub)scale separately?	Internal consistency statistic calculated for each subscale separately			Internal consistency statistic NOT calculated for each subscale separately
8	Were there any important flaws in the design or methods of the study?	No other important methodological flaws in the design or execution of the study		Other minor methodological flaws in the design or execution of the study	Other important methodological flaws in the design or execution of the study
	Statistical methods				
9	for Classical Test Theory (CTT), continuous scores: Was Cronbach's alpha calculated?	Cronbach's alpha calculated		Only item-total correlations calculated	No Cronbach's alpha and no item-total correlations calculated
10	for CTT, dichotomous scores: Was Cronbach's alpha or KR-20 calculated?	Cronbach's alpha or KR-20 calculated		Only item-total correlations calculated	No Cronbach's alpha or KR-20 and no item-total correlations calculated
11	for IRT: Was a goodness of fit statistic at a global level calculated? E.g. χ^2, reliability coefficient of estimated latent trait value (index of (subject or item) separation)	Goodness of fit statistic at a global level calculated			Goodness of fit statistic at a global level NOT calculated

NB. Item 1 is used to determine whether internal consistency is relevant for the instrument under study. It is not used to rate the quality of the study.

**Box B. Reliability: relative measures (including test-retest reliability, inter-rater reliability and intra-rater reliability)**					
		**excellent**	**good**	**fair**	**poor**
	Design requirements				
1	Was the percentage of missing items given?	Percentage of missing items described	Percentage of missing items NOT described		
2	Was there a description of how missing items were handled?	Described how missing items were handled	Not described but it can be deduced how missing items were handled	Not clear how missing items were handled	
3	Was the sample size included in the analysis adequate?	Adequate sample size (≥100)	Good sample size (50-99)	Moderate sample size (30-49)	Small sample size (<30)
4	Were at least two measurements available?	At least two measurements			Only one measurement
5	Were the administrations independent?	Independent measurements	Assumable that the measurements were independent	Doubtful whether the measurements were independent	measurements NOT independent
6	Was the time interval stated?	Time interval stated		Time interval NOT stated	
7	Were patients stable in the interim period on the construct to be measured?	Patients were stable (evidence provided)	Assumable that patients were stable	Unclear if patients were stable	Patients were NOT stable
8	Was the time interval appropriate?	Time interval appropriate		Doubtful whether time interval was appropriate	Time interval NOT appropriate
9	Were the test conditions similar for both measurements? e.g. type of administration, environment, instructions	Test conditions were similar (evidence provided)	Assumable that test conditions were similar	Unclear if test conditions were similar	Test conditions were NOT similar

10	Were there any important flaws in the design or methods of the study?	No other important methodological flaws in the design or execution of the study		Other minor methodological flaws in the design or execution of the study	Other important methodological flaws in the design or execution of the study
Statistical methods					
11	for continuous scores: Was an intraclass correlation coefficient (ICC) calculated?	ICC calculated and model or formula of the ICC is described	ICC calculated but model or formula of the ICC not described or not optimal. Pearson or Spearman correlation coefficient calculated with evidence provided that no systematic change has occurred	Pearson or Spearman correlation coefficient calculated WITHOUT evidence provided that no systematic change has occurred or WITH evidence that systematic change has occurred	No ICC or Pearson or Spearman correlations calculated
12	for dichotomous/nominal/ordinal scores: Was kappa calculated?	Kappa calculated			Only percentage agreement calculated
13	for ordinal scores: Was a weighted kappa calculated?	Weighted Kappa calculated		Unweighted Kappa calculated	Only percentage agreement calculated
14	for ordinal scores: Was the weighting scheme described? e.g. linear, quadratic	Weighting scheme described	Weighting scheme NOT described		

Box C. Measurement error: absolute measures

		excellent	good	fair	poor
Design requirements					
1	Was the percentage of missing items given?	Percentage of missing items described	Percentage of missing items NOT described		
2	Was there a description of how missing items were handled?	Described how missing items were handled	Not described but it can be deduced how missing items were handled	Not clear how missing items were handled	
3	Was the sample size included in the analysis adequate?	Adequate sample size (≥100)	Good sample size (50-99)	Moderate sample size (30-49)	Small sample size (<30)
4	Were at least two measurements available?	At least two measurements			Only one measurement
5	Were the administrations independent?	Independent measurements	Assumable that the measurements were independent	Doubtful whether the measurements were independent	measurements NOT independent
6	Was the time interval stated?	Time interval stated		Time interval NOT stated	
7	Were patients stable in the interim period on the construct to be measured?	Patients were stable (evidence provided)	Assumable that patients were stable	Unclear if patients were stable	Patients were NOT stable
8	Was the time interval appropriate?	Time interval appropriate		Doubtful whether time interval was appropriate	Time interval NOT appropriate
9	Were the test conditions similar for both measurements? e.g. type of administration, environment, instructions	Test conditions were similar (evidence provided)	Assumable that test conditions were similar	Unclear if test conditions were similar	Test conditions were NOT similar

10	Were there any important flaws in the design or methods of the study?	No other important methodological flaws in the design or execution of the study		Other minor methodological flaws in the design or execution of the study	Other important methodological flaws in the design or execution of the study
	Statistical methods				
11	for CTT: Was the Standard Error of Measurement (SEM), Smallest Detectable Change (SDC) or Limits of Agreement (LoA) calculated?	SEM, SDC, or LoA calculated	Possible to calculate LoA from the data presented		SEM calculated based on Cronbach's alpha, or on SD from another population

Box D. Content validity (including face validity)

		excellent	good	fair	poor
	General requirements				
1	Was there an assessment of whether all items refer to relevant aspects of the construct to be measured?	Assessed if all items refer to relevant aspects of the construct to be measured		Aspects of the construct to be measured poorly described AND this was not taken into consideration	NOT assessed if all items refer to relevant aspects of the construct to be measured
2	Was there an assessment of whether all items are relevant for the study population? (e.g. age, gender, disease characteristics, country, setting)	Assessed if all items are relevant for the study population in adequate sample size (≥10)	Assessed if all items are relevant for the study population in moderate sample size (5-9)	Assessed if all items are relevant for the study population in small sample size (<5)	NOT assessed if all items are relevant for the study population OR target population not involved
3	Was there an assessment of whether all items are relevant for the purpose of the measurement instrument? (discriminative, evaluative, and/or predictive)	Assessed if all items are relevant for the purpose of the application	Purpose of the instrument was not described but assumed	NOT assessed if all items are relevant for the purpose of the application	
4	Was there an assessment of whether all items together comprehensively reflect the construct to be measured?	Assessed if all items together comprehensively reflect the construct to be measured		No theoretical foundation of the construct and this was not taken into consideration	NOT assessed if all items together comprehen-sively reflect the construct to be measured
5	Were there any important flaws in the design or methods of the study?	No other important methodological flaws in the design or execution of the study		Other minor methodological flaws in the design or execution of the study	Other important methodological flaws in the design or execution of the study

Box E. Structural validity	excellent	good	fair	poor
1 Does the scale consist of effect indicators, i.e. is it based on a reflective model?				
Design requirements				
2 Was the percentage of missing items given?	Percentage of missing items described	Percentage of missing items NOT described		
3 Was there a description of how missing items were handled?	Described how missing items were handled	Not described but it can be deduced how missing items were handled	Not clear how missing items were handled	
4 Was the sample size included in the analysis adequate?	7* #items and ≥100	5* #items and ≥100 OR 5-7* #items but <100	5* #items but <100	<5* #items
5 Were there any important flaws in the design or methods of the study?	No other important methodological flaws in the design or execution of the study		Other minor methodological flaws in the design or execution of the study (e.g. rotation method not described)	Other important methodological flaws in the design or execution of the study (e.g. inappropriate rotation method)

Statistical methods				
6 for CTT: Was exploratory or confirmatory factor analysis performed?	Exploratory or confirmatory factor analysis performed and type of factor analysis appropriate in view of existing information	Exploratory factor analysis performed while confirmatory would have been more appropriate		No exploratory or confirmatory factor analysis performed
7 for IRT: Were IRT tests for determining the (uni-) dimensionality of the items performed?	IRT test for determining (uni)dimension-ality performed			IRT test for determining (uni)dimension-ality NOT performed

Box F. Hypotheses testing	excellent	good	fair	Poor
Design requirements				
1 Was the percentage of missing items given?	Percentage of missing items described	Percentage of missing items NOT described		
2 Was there a description of how missing items were handled?	Described how missing items were handled	Not described but it can be deduced how missing items were handled	Not clear how missing items were handled	
3 Was the sample size included in the analysis adequate?	Adequate sample size (≥100 per analysis)	Good sample size (50-99 per analysis)	Moderate sample size (30-49 per analysis)	Small sample size (<30 per analysis)

4 Were hypotheses regarding correlations or mean differences formulated a priori (i.e. before data collection)?	Multiple hypotheses formulated a priori	Minimal number of hypotheses formulate a priori	Hypotheses vague or not formulated but possible to deduce what was expected	Unclear what was expected
5 Was the expected *direction* of correlations or mean differences included in the hypotheses?	Expected direction of the correlations or differences stated	Expected direction of the correlations or differences NOT stated		
6 Was the expected absolute or relative *magnitude* of correlations or mean differences included in the hypotheses?	Expected magnitude of the correlations or differences stated	Expected magnitude of the correlations or differences NOT stated		
7 for convergent validity: Was an adequate description provided of the comparator instrument(s)?	Adequate description of the constructs measured by the comparator instrument(s)	Adequate description of most of the constructs measured by the comparator instrument(s)	Poor description of the constructs measured by the comparator instrument(s)	NO description of the constructs measured by the comparator instrument(s)
8 for convergent validity: Were the measurement properties of the comparator instrument(s) adequately described?	Adequate measurement properties of the comparator instrument(s) in a population similar to the study population	Adequate measurement properties of the comparator instrument(s) but not sure if these apply to the study population	Some information on measurement properties (or a reference to a study on measurement properties) of the comparator instrument(s) in any study population	No information on the measurement properties of the comparator instrument(s)

9 Were there any important flaws in the design or methods of the study?	No other important methodological flaws in the design or execution of the study		Other minor methodological flaws in the design or execution of the study (e.g. only data presented on a comparison with an instrument that measures another construct)	Other important methodological flaws in the design or execution of the study
Statistical methods				
10 Were design and statistical methods adequate for the hypotheses to be tested?	Statistical methods applied appropriate	Assumable that statistical methods were appropriate, e.g. Pearson correlations applied, but distribution of scores or mean (SD) not presented	Statistical methods applied NOT optimal	Statistical methods applied NOT appropriate

**Box G. Cross-cultural validity**				
	**excellent**	**good**	**fair**	**poor**
Design requirements				
1 Was the percentage of missing items given?	Percentage of missing items described	Percentage of missing items NOT described		
2 Was there a description of how missing items were handled?	Described how missing items were handled	Not described but it can be deduced how missing items were handled	Not clear how missing items were handled	

3	Was the sample size included in the analysis adequate?	CTT: 7* #items and ≥100<br>IRT: ≥200 per group	CTT: 5* #items and ≥100 OR 5-7* #items but <100<br>IRT: ≥200 in 1 group and 100-199 in 1 group	CTT: 5* #items but <100<br>IRT: 100-199 per group	CTT: <5* #items<br>IRT: (<100 in 1 or both groups
4	Were both the original language in which the HR-PRO instrument was developed, and the language in which the HR-PRO instrument was translated described?	Both source language and target language described			Source language NOT known
5	Was the expertise of the people involved in the translation process adequately described? e.g. expertise in the disease(s) involved, expertise in the construct to be measured, expertise in both languages	Expertise of the translators described with respect to disease, construct, and language	Expertise of the translators with respect to disease or construct poor or not described	Expertise of the translators with respect to language not described	
6	Did the translators work independently from each other?	Translators worked independent	Assumable that the translators worked independent	Unclear whether translators worked independent	Translators worked NOT independent
7	Were items translated forward and backward?	Multiple forward and multiple backward translations	Multiple forward translations but one backward translation	One forward and one backward translation	Only a forward translation
8	Was there an adequate description of how differences between the original and translated versions were resolved?	Adequate description of how differences between translators were resolved	Poorly or NOT described how differences between translators were resolved		
9	Was the translation reviewed by a committee (e.g. original developers)?	Translation reviewed by a committee (involving other people than the translators, e.g. the original developers)	Translation NOT reviewed by (such) a committee		
10	Was the HR-PRO instrument pre-tested (e.g. cognitive interviews) to check interpretation, cultural relevance of the translation, and ease of comprehension?	Translated instrument pre-tested in the target population	Translated instrument pre-tested, but unclear if this was done in the target population	Translated instrument pre-tested, but NOT in the target population	Translated instrument NOT pre-tested
11	Was the sample used in the pre-test adequately described?	Sample used in the pre-test adequately described		Sample used in the pre-test NOT (adequately) described	
12	Were the samples similar for all characteristics except language and/or cultural background?	Shown that samples were similar for all characteristics except language /culture	Stated (but not shown) that samples were similar for all characteristics except language /culture	Unclear whether samples were similar for all characteristics except language /culture	Samples were NOT similar for all characteristics except language /culture
13	Were there any important flaws in the design or methods of the study?	No other important methodological flaws in the design or execution of the study		Other minor methodological flaws in the design or execution of the study	Other important methodological flaws in the design or execution of the study

	excellent	good	fair	poor
Statistical methods				
14 for CTT: Was confirmatory factor analysis performed?	Multiple-group confirmatory factor analysis performed			Multiple-group confirmatory factor analysis NOT performed
15 for IRT: Was differential item function (DIF) between language groups assessed?	DIF between language groups assessed			DIF between language groups NOT assessed

Box H. Criterion validity

	excellent	good	fair	poor
Design requirements				
1 Was the percentage of missing items given?	Percentage of missing items described	Percentage of missing items NOT described		
2 Was there a description of how missing items were handled?	Described how missing items were handled	Not described but it can be deduced how missing items were handled	Not clear how missing items were handled	
3 Was the sample size included in the analysis adequate?	Adequate sample size (≥100)	Good sample size (50-99)	Moderate sample size (30-49)	Small sample size (<30)
4 Can the criterion used or employed be considered as a reasonable 'gold standard'?	Criterion used can be considered an adequate 'gold standard' (evidence provided)	No evidence provided, but assumable that the criterion used can be considered an adequate 'gold standard'	Unclear whether the criterion used can be considered an adequate 'gold standard'	Criterion used can NOT be considered an adequate 'gold standard'

	excellent	good	fair	poor
5 Were there any important flaws in the design or methods of the study?	No other important methodological flaws in the design or execution of the study		Other minor methodological flaws in the design or execution of the study	Other important methodological flaws in the design or execution of the study
Statistical methods				
6 for continuous scores: Were correlations, or the area under the receiver operating curve calculated?	Correlations or AUC calculated			Correlations or AUC NOT calculated
7 for dichotomous scores: Were sensitivity and specificity determined?	Sensitivity and specificity calculated			Sensitivity and specificity NOT calculated

Box I. Responsiveness

	excellent	good	fair	poor
Design requirements				
1 Was the percentage of missing items given?	Percentage of missing items described	Percentage of missing items NOT described		
2 Was there a description of how missing items were handled?	Described how missing items were handled	Not described but it can be deduced how missing items were handled	Not clear how missing items were handled	
3 Was the sample size included in the analysis adequate?	Adequate sample size (≥100)	Good sample size (50-99)	Moderate sample size (30-49)	Small sample size (<30)
4 Was a longitudinal design with at least two measurement used?	Longitudinal design used			No longitudinal design used
5 Was the time interval stated?	Time interval adequately described			Time interval NOT described

6 If anything occurred in the interim period (e.g. intervention, other relevant events), was it adequately described?	Anything that occurred during the interim period (e.g. treatment) adequately described	Assumable what occurred during the interim period	Unclear or NOT described what occurred during the interim period	
7 Was a proportion of the patients changed (i.e. improvement or deterioration)?	Part of the patients were changed (evidence provided)	NO evidence provided, but assumable that part of the patients were changed	Unclear if part of the patients were changed	Patients were NOT changed
Design requirements for hypotheses testing				
For constructs for which a gold standard was not available:				
8 Were hypotheses about changes in scores formulated a priori (i.e. before data collection)?	Hypotheses formulated a priori		Hypotheses vague or not formulated but possible to deduce what was expected	Unclear what was expected
9 Was the expected *direction* of correlations or mean differences of the change scores of HR-PRO instruments included in these hypotheses?	Expected direction of the correlations or differences stated	Expected direction of the correlations or differences NOT stated		
10 Were the expected absolute or relative *magnitude* of correlations or mean differences of the change scores of HR-PRO instruments included in these hypotheses?	Expected magnitude of the correlations or differences stated	Expected magnitude of the correlations or differences NOT stated		
11 Was an adequate description provided of the comparator instrument(s)?	Adequate description of the constructs measured by the comparator instrument(s)		Poor description of the constructs measured by the comparator instrument(s)	NO description of the constructs measured by the comparator instrument(s)
12 Were the measurement properties of the comparator instrument(s) adequately described?	Adequate measurement properties of the comparator instrument(s) in a population similar to the study population	Adequate measurement properties of the comparator instrument(s) but not sure if these apply to the study population	Some information on measurement properties (or a reference to a study on measurement properties) of the comparator instrument(s) in any study population	NO information on the measurement properties of the comparator instrument(s)
13 Were there any important flaws in the design or methods of the study?	No other important methodological flaws in the design or execution of the study		Other minor methodological flaws in the design or execution of the study (e.g. only data presented on a comparison with an instrument that measures another construct)	Other important methodological flaws in the design or execution of the study
Statistical methods				
14 Were design and statistical methods adequate for the hypotheses to be tested?	Statistical methods applied appropriate		Statistical methods applied NOT optimal	Statistical methods applied NOT appropriate

Design requirement for comparison to a gold standard					
	For constructs for which a gold standard was available:				
15	Can the criterion for change be considered as a reasonable gold standard?	Criterion used can be considered an adequate 'gold standard' (evidence provided)	No evidence provided, but assumable that the criterion used can be considered an adequate 'gold standard'	Unclear whether the criterion used can be considered an adequate 'gold standard'	Criterion used can NOT be considered an adequate 'gold standard'
16	Were there any important flaws in the design or methods of the study?	No other important methodological flaws in the design or execution of the study		Other minor methodological flaws in the design or execution of the study	Other important methodological flaws in the design or execution of the study
Statistical methods					
17	for continuous scores: Were correlations between change scores, or the area under the Receiver Operator Curve (ROC) curve calculated?	Correlations or Area under the ROC Curve (AUC) calculated			Correlations or AUC NOT calculated
18	for dichotomous scales: Were sensitivity and specificity (changed versus not changed) determined?	Sensitivity and specificity calculated			Sensitivity and specificity NOT calculated

Interpretability

We recommend to use the Interpretability box to extract all information on the interpretability issues described in this box of the instruments under study from the included articles.

**Box Interpretability**	
Percentage of missing items	
Description of how missing items were handled	
Distribution of the (total) scores	
Percentage of the respondents who had the lowest possible (total) score	
Percentage of the respondents who had the highest possible (total) score	
Scores and change scores (i.e. means and SD) for relevant (sub) groups, e.g. for normative groups, subgroups of patients, or the general population	
Minimal Important Change (MIC) or Minimal Important Difference (MID)	

Generalizability

We recommend to use the Generalizability box to extract data on the characteristics of the study populations and sampling procedures of the included studies.

**Box Generalisability**	
Median or mean age (with standard deviation or range)	
Distribution of sex	
Important disease characteristics (e.g. severity, status, duration) and description of treatment	
Setting(s) in which the study was conducted (e.g. general population, primary care or hospital/rehabilitation care)	
Countries in which the study was conducted	
Language in which the HR-PRO instrument was evaluated	
Method used to select patients (e.g. convenience, consecutive, or random)	
Percentage of missing responses (response rate)	

Supplemental Information 2: Parent-Report Camouflaging Autistic Traits Questionnaire (CAT-Q)

Parent-Report Camouflaging Autistic Traits Questionnaire

Please read each statement below and choose the answer that best fits your perception of your child's experiences during social interactions.

Strongly Disagree (1)	Disagree (2)	Somewhat Disagree (3)	Neither Agree nor Disagree (4)	Somewhat Agree (5)	Agree (6)	Strongly Agree (7)

1. When my child is interacting with someone, they deliberately copy the other person's body language or facial expressions
2. My child monitors their body language or facial expressions so that they appear relaxed
3. My child rarely feels the need to put on an act in order to get through a social situation*
4. My child has developed a script to follow in social situations (for example, a list of questions or topics of conversation)
5. My child will repeat phrases that they have heard others say in the exact same way that they first heard them
6. My child adjusts their body language or facial expressions so that they appear interested by the person they are interacting with
7. In social situations, my child is 'performing' rather than being themselves
8. In my child's social interactions, they use behaviours that they have learned from watching other people interacting
9. My child always thinks about the impression they make on other people
10. My child needs the support of other people in order to socialise
11. My child practices their facial expressions and body language to make sure they look natural
12. My child doesn't feel the need to make eye contact with other people if they don't want to*
13. My child has to force themselves to interact with people when they are in social situations
14. My child has tried to improve their understanding of social skills by watching other people

15. My child monitors their body language or facial expressions so that they appear interested by the person they are interacting with
16. When in social situations, my child tries to find ways to avoid interacting with others
17. My child has researched the rules of social interactions (for example, by studying psychology or reading books on human behaviour) to improve their own social skills
18. My child is always aware of the impression they make on other people
19. My child feels free to be themselves when they are with other people*
20. My child learns how people use their bodies and faces to interact by watching television or films, or by reading fiction
21. My child adjusts their body language or facial expressions so that they appear relaxed
22. When my child is talking to other people, the conversation flows naturally*
23. My child has spent time learning social skills from television shows and films, and tries to use these in their interactions
24. In social interactions, my child does not pay attention to what their face or body are doing*
25. In social situations, my child feels like they are pretending to be 'normal'

Scoring:

All items are scored 1-7, with higher scores reflecting greater camouflaging. Items with an asterisk (*) should be reverse scored.

Factors:

Compensation = 1, 4, 5, 8, 11, 14, 17, 20, 23

Masking = 2, 6, 9, 12, 15, 18, 21, 24

Assimilation = 3, 7, 10, 13, 16, 19, 22, 25

Hannon, Mandy & Hull, under review
