# Supplementary material for: A comparison of methods for measuring camouflaging in autism
Source: Autism Res. 2022 Nov 24;16(1):12–29. doi: 10.1002/aur.2850 (PMC10099783; doi:10.1002/aur.2850)
Supplement: Supplementary file 2 — Table S1 Additional papers identified during the literature search. [file AUR-16-12-s001.docx]

Supplementary Table 1: Additional papers identified during the literature search

| **Name of Measure** | **Internal consistency** | **Reliability** | **Measurement error** | **Content validity** | **Structural validity** | **Hypothesis testing** | **Cross-cultural validity / Measurement Invariance** | **Criterion validity** | **Responsiveness** |
| --- | --- | --- | --- | --- | --- | --- | --- | --- | --- |
| **CAT-Q** |  |  |  |  |  |  |  |  |  |
| Beck et al. (2020) | * | * | * | * | * |  |  |  |  |
| Belcher et al., (2022) | * | * | * | * | * |  |  |  |  |
| Bowri et al. (2021) |  | * | * | * | * |  |  |  |  |
| Cage & Troxell-Whitman (2020) |  | * | * | * | * |  |  |  |  |
| Cassidy et al. (2020) |  | * | * | * | * |  |  |  |  |
| Cassidy et al. (2021) |  | * | * | * | * |  |  |  |  |
| Cook et al. (2021) |  | * | * | * | * |  |  |  |  |
| Dell’Osso et al. (2022) |  |  |  | * | * |  |  |  |  |
| Hull et al. (2020) |  | * | * | * | * |  |  |  |  |
| Hull, Petrides & Mandy (2020) |  | * | * | * | * |  |  |  |  |
| Jorgenson et al. (2020) |  | * | * | * | * |  |  |  |  |
| McQuaid, Lee & Wallace (2022) |  | * | * | * | * |  |  |  |  |
| Perry et al. (2022) |  | * | * | * | * |  |  |  |  |
| Robinson, Hull & Petrides (2020) |  | * | * | * | * |  |  |  |  |
| Scheerer et al. 2020 | * | * | * | * | * |  |  |  |  |
| Walsh et al. (2021) | * | * | * | * | * |  |  |  |  |
| **The Livingston Method** |  |  |  |  |  |  |  |  |  |
| Corbett et al. (2021)** |  |  |  |  |  |  |  |  |  |

*These ratings reflect the authors reference to the original paper that measured these variables. Therefore, these variables were not assessed within the paper.

**Note that all the ratings for the repetition of the Livingston method do not refer back to the original paper. This is because the method itself is specific to the sample, i.e. group allocation is relative to median split of the sample used.

Beck, J. S., Lundwall, R. A., Gabrielsen, T., Cox, J. C., & South, M. (2020). Looking Good but Feeling Bad: “Camouflaging” Behaviors and Mental Health in Women with Autistic Traits. Autism, 24, 809-821.

<https://doi.org/10.1177/1362361320912147>

Belcher, H. L., Morein-Zamir, S., Mandy, W., & Ford, R. M. (2022). Camouflaging intent, first impressions, and age of ASC diagnosis in autistic men and women. *Journal of Autism and Developmental Disorders*, *52*(8), 3413-3426.

<https://doi.org/10.1007/s10803-021-05221-3>

Bernardin, C. J., Mason, E., Lewis, T., & Kanne, S. (2021). “You must become a chameleon to survive”: adolescent experiences of camouflaging. *Journal of Autism and Developmental Disorders*, *51*(12), 4422-4435.

<https://doi.org/10.1007/s10803-021-04912-1>

Bowri, M., Hull, L., Allison, C., Smith, P., Baron-Cohen, S., Lai, M. C., & Mandy, W. (2021). Demographic and psychological predictors of alcohol use and misuse in autistic adults. *Autism*, *25*(5), 1469-1480.

<https://doi.org/10.1177/1362361321992668>

Cage, E., & Troxell-Whitman, Z. (2020). Understanding the relationships between autistic identity, disclosure, and camouflaging. *Autism in Adulthood*, *2*(4), 334-338.

<https://doi.org/10.1007/s10803-018-03878-x>

Cassidy, S. A., Bradley, L., Cogger-Ward, H., & Rodgers, J. (2021). Development and validation of the suicidal behaviours questionnaire-autism spectrum conditions in a community sample of autistic, possibly autistic and non-autistic adults. *Molecular autism*, *12*(1), 1-22.

<https://doi.org/10.1186/s13229-021-00449-3>

Cassidy, S. A., Gould, K., Townsend, E., Pelton, M., Robertson, A. E., & Rodgers, J. (2020). Is camouflaging autistic traits associated with suicidal thoughts and behaviours? Expanding the interpersonal psychological theory of suicide in an undergraduate student sample. *Journal of autism and developmental disorders*, *50*(10), 3638-3648.

<https://doi.org/10.1007/s10803-019-04323-3>

Cook, J., Hull, L., Crane, L., & Mandy, W. (2021). Camouflaging in autism: A systematic review. Clinical Psychology Review, 89, 102080.

<https://doi.org/10.1016/j.cpr.2021.102080>

Corbett, B. A., Schwartzman, J. M., Libsack, E. J., Muscatello, R. A., Lerner, M. D., Simmons, G. L., & White, S. W. (2021). Camouflaging in autism: Examining sex‐based and compensatory models in social cognition and communication. *Autism research*, *14*(1), 127-142.

<https://doi.org/10.1002/aur.2440>

Dell'Osso, L., Cremone, I. M., Muti, D., Massimetti, G., Lorenzi, P., Carmassi, C., & Carpita, B. (2022). Validation of the Italian version of the Camouflaging Autistic Traits Questionnaire (CAT-Q) in a University population. *Comprehensive Psychiatry*, *114*, 152295.

<https://doi.org/10.1016/j.comppsych.2022.152295>

Hull, L., Lai, M. C., Baron-Cohen, S., Allison, C., Smith, P., Petrides, K. V., & Mandy, W. (2020). Gender differences in self-reported camouflaging in autistic and non-autistic adults. *Autism*, *24*(2), 352-363.

<https://doi.org/10.1177/1362361319864804>

Hull, L., Petrides, K. V., & Mandy, W. (2020). Cognitive predictors of self‐reported camouflaging in autistic adolescents. *Autism Research*, *14*(3), 523-532.

<https://doi.org/10.1002/AUR.2407>

Jorgenson, C., Lewis, T., Rose, C., & Kanne, S. (2020). Social camouflaging in autistic and neurotypical adolescents: A pilot study of differences by sex and diagnosis. *Journal of Autism and Developmental Disorders*, *50*(12), 4344-4355.

<https://doi.org/10.1007/s10803-020-04491-7>

Lai, M. C., Lombardo, M. V., Chakrabarti, B., Ruigrok, A. N., Bullmore, E. T., Suckling, J., ... & Baron-Cohen, S. (2019). Neural self-representation in autistic women and association with ‘compensatory camouflaging’. *Autism*, *23*, 1210-1223.

<https://doi.org/10.1177/1362361318807159>

Robinson, E., Hull, L., & Petrides, K. V. (2020). Big Five Model and Trait Emotional Intelligence in Camouflaging Behaviours in Autism. *Personality and Individual Differences*, *152*.

<https://doi.org/10.1016/j.paid.2019.109565>

McQuaid, G. A., Lee, N. R., & Wallace, G. L. (2022). Camouflaging in autism spectrum disorder: examining the roles of sex, gender identity, and diagnostic timing. *Autism*, *26*(2), 552-559.

<https://doi.org/10.1177/13623613211042131>

Perry, E., Mandy, W., Hull, L., & Cage, E. (2022). Understanding camouflaging as a response to autism-related stigma: A social identity theory approach. *Journal of Autism and Developmental Disorders*, *52*(2), 800-810.

<https://doi.org/10.1007/s10803-021-04987-w>.

Scheerer, N. E., Aime, H., Boucher, T., & Iarocci, G. (2020). The association between self-reported camouflaging of autistic traits and social competence in nonautistic young adults. *Autism in Adulthood*, *2*(4), 298-306.

<https://doi.org/10.1089/aut.2019.0062>

Walsh, M. J., Pagni, B., Monahan, L., Delaney, S., Smith, C. J., Baxter, L., & Braden, B. B. (2021). Sex-Related Neurocircuitry Supporting Camouflaging in Adults with Autism: Female Protection Insights. *bioRxiv*.

<https://doi.org/10.1101/2021.11.03.466990>
